# Yield and Economic Performance of Organic and Conventional Cotton-Based Farming Systems – Results from a Field Trial in India

**DOI:** 10.1371/journal.pone.0081039

**Published:** 2013-12-04

**Authors:** Dionys Forster, Christian Andres, Rajeev Verma, Christine Zundel, Monika M. Messmer, Paul Mäder

**Affiliations:** 1 International Division, Research Institute of Organic Agriculture (FiBL), Frick, Switzerland; 2 Research Division, bioRe Association, Kasrawad, Madhya Pradesh, India; 3 Ecology Group, Federal Office for Agriculture (FOAG), Bern, Switzerland; 4 Soil Sciences Division, Research Institute of Organic Agriculture (FiBL), Frick, Switzerland; CIRAD, France

## Abstract

The debate on the relative benefits of conventional and organic farming systems has in recent time gained significant interest. So far, global agricultural development has focused on increased productivity rather than on a holistic natural resource management for food security. Thus, developing more sustainable farming practices on a large scale is of utmost importance. However, information concerning the performance of farming systems under organic and conventional management in tropical and subtropical regions is scarce. This study presents agronomic and economic data from the conversion phase (2007–2010) of a farming systems comparison trial on a Vertisol soil in Madhya Pradesh, central India. A cotton-soybean-wheat crop rotation under biodynamic, organic and conventional (with and without Bt cotton) management was investigated. We observed a significant yield gap between organic and conventional farming systems in the 1^st^ crop cycle (cycle 1: 2007–2008) for cotton (−29%) and wheat (−27%), whereas in the 2^nd^ crop cycle (cycle 2: 2009–2010) cotton and wheat yields were similar in all farming systems due to lower yields in the conventional systems. In contrast, organic soybean (a nitrogen fixing leguminous plant) yields were marginally lower than conventional yields (−1% in cycle 1, −11% in cycle 2). Averaged across all crops, conventional farming systems achieved significantly higher gross margins in cycle 1 (+29%), whereas in cycle 2 gross margins in organic farming systems were significantly higher (+25%) due to lower variable production costs but similar yields. Soybean gross margin was significantly higher in the organic system (+11%) across the four harvest years compared to the conventional systems. Our results suggest that organic soybean production is a viable option for smallholder farmers under the prevailing semi-arid conditions in India. Future research needs to elucidate the long-term productivity and profitability, particularly of cotton and wheat, and the ecological impact of the different farming systems.

## Introduction

The green revolution has brought about a series of technological achievements in agricultural production, particularly in Asia. Worldwide cereal harvests tripled between 1950 and 2000, making it possible to provide enough dietary calories for a world population of six billion by the end of the 20th century [1]. So far, global agricultural development has rather focused on increased productivity than on a more holistic natural resource management for food security and sovereignty. The increase in food production has been accompanied by a multitude of challenges and problems such as the exploitation and deterioration of natural resources, i.e. loss of soil fertility, strong decline of agro-biodiversity, pollution of water [2], [3], and health problems associated with the use of synthetic plant protection products [4]. At present, more comprehensive system-oriented approaches are gaining momentum and are expected to better address the difficult issues associated with the complexity of farming systems in different locations and cultures [5].

The concept of organic agriculture builds on the idea of the efficient use of locally available resources as well as the usage of adapted technologies (e.g. soil fertility management, closing of nutrient cycles as far as possible, control of pests and diseases through management and natural antagonists). It is based on a system-oriented approach and can be a promising option for sustainable agricultural intensification in the tropics, as it may offer several potential benefits [6]–[11] such as: (i) A greater yield stability, especially in risk-prone tropical ecosystems, (ii) higher yields and incomes in traditional farming systems, once they are improved and the adapted technologies are introduced, (iii) an improved soil fertility and long-term sustainability of farming systems, (iv) a reduced dependence of farmers on external inputs, (v) the restoration of degraded or abandoned land, (vi) the access to attractive markets through certified products, and (vii) new partnerships within the whole value chain, as well as a strengthened self-confidence and autonomy of farmers. Critics contend that organic agriculture is associated with low labor productivity and high production risks [1], [12]–[14], as well as high certification costs for smallholders [15]. However, the main criticism reflected in the scientific literature is the claim that organic agriculture is not able to meet the world's growing food demand, as yields are on average 20% to 25% lower than in conventional agriculture [16], [17]. It should however be taken into account, that yield deviations among different crops and regions can be substantial depending on system and site characteristics [16], [17]. In a meta-analysis by Seufert *et al*. [17] it is shown that yields in organic farming systems with good management practices can nearly match conventional yields, whereas under less favorable conditions they cannot. However, Reganold [18] pointed out that productivity is not the only goal that must be met in order for agriculture to be considered sustainable: The maintenance or enhancement of soil fertility and biodiversity, while minimizing detrimental effects on the environment and the contribution to the well-being of farmers and their communities are equally important as the above mentioned productivity goals. Farming systems comparison trials should thus - besides agronomic determinants - also consider ecological and economic factors over a longer period. These trials are inherently difficult due to the many elements the farming systems are comprised of, thus necessitating holistic research approaches in order to make comparisons possible [19].

Results from various farming systems comparison trials between organic and conventional management have shown, that even though yields may be slightly lower, organic farming systems exhibit several ecological and economic advantages, particularly long-term improvement of soil fertility [20]–[25]. However, most of the data has been obtained from trials in the temperate zones [20]–[26]. The little data available under tropical and subtropical conditions [9], [27]–[29] calls for more long-term farming systems comparison trials to provide a better basis for decision making in these regions [17]. To address this issue, the Research Institute of Organic Agriculture (FiBL) has set up three farming systems comparison trials in Kenya, India and Bolivia, thereby encompassing different cropping systems and ethnologies. The main objective of these trials is to collect solid agronomic and socio-economic data on major organic and conventional agricultural production systems in the selected project regions. These trials will contribute to close the existing knowledge gap regarding the estimation of profitability of organic agriculture in developing countries (http://www.systems-comparison.fibl.org/). This paper presents results from cotton-based farming systems in India.

India is the second largest producer (after China) of cotton lint worldwide [30]. Cotton is a very important cash crop for smallholder farmers, but also one of the most exigent crops in terms of agrochemical inputs which are responsible for adverse effects on human health and the environment [27]. Genetically modified (GM) cotton hybrids carrying a gene of *Bacillus thuringiensis* (Bt) for protection against bollworm (*Helicoverpa* spp.) attack, have spread rapidly after their official introduction to India in 2002 [31], [32]. By 2012, 7 million farmers cultivating 93% of India's total cotton area had adopted Bt cotton technology [32], [33]. This high adoption rate might be attributed to the high pressure caused by cotton bollworms, and associated reductions in pesticide use upon the introduction of Bt cotton technology in India [34], [35]. However, the discussion about the impacts of Bt cotton adoption remains highly controversial [36], [37]. Giving focus to yields, advocates of Bt cotton claim that the technology has led to an increase in productivity of up to 60% [38]–[40] and in some cases even “near 100%” [41]. Opponents of Bt cotton on the other hand attributed the yield gains, compared to the pre-Bt period, to other factors. These include (i) the increase of the area under cotton cultivation, (ii) the shift from traditional diploid cotton (*G. arboreum, G. herbaceum* which accounted for 28% of total cotton area in 2000) to tetraploid *G. hirsutum* species [42] and the widespread adoption of hybrid seeds, (iii) the increased use of irrigation facilities, (iv) the introduction of new pesticides with novel action (e.g. Imidacloprid seed treatment), and (v) the increased use of fertilizers in Bt cotton cultivation [43], [44]. Critics of Bt crops also stress uncertainties concerning the impact of the technology on human health [45] and on non-target organisms [46], as well as the higher costs of Bt seeds [34], [47].

While some argue that GM crops in general can contribute significantly to sustainable development at the global level [33], [48], others state that there is no scientific support for this claim [49]. Considering economic benefits of Bt cotton, the same controversy prevails: Advocates claim sustainable socio-economic benefits and associated social development [33], [36], while opponents claim Bt cotton to be responsible for farmer debt [50], thereby contributing to India's notoriety for farmers' suicides [37], [51], a linkage which has been criticized as reductionist and invalid [52]. However, comparisons are mainly drawn between Bt and non-Bt cotton under conventional management in high-input farming systems. Organic cotton production systems - holding a minor percentage of the cotton growing area in India - are often neglected, and little information exists on the productivity and profitability of organic farming in India [53]. However, organic cotton production is slowly gaining momentum in the global cotton market [27]. GM cultivars are not compatible with the guidelines of organic agriculture [54]. Therefore, organic cotton producers have to refrain from Bt cotton hybrids. In addition, organic producers and processors have to take all possible measures to avoid contamination with Bt cotton in order not to lose organic certification.

While organic farming systems have attracted considerable interest of the scientific community [16], [17], [21], [26], biodynamic farming systems are less common and little investigated. The biodynamic agricultural movement started in the early 1920s in Europe [55] and developed the international certification organization and label DEMETER. In India, the biodynamic movement started in the early 1990s (www.biodynamics.in). Preparations made from manure, minerals and herbs are used in very small quantities to activate and harmonize soil processes, to strengthen plant health and to stimulate processes of organic matter decomposition. Most biodynamic farms encompass ecological, social and economic sustainability and many of them work in cooperatives. One of the first initiatives in India was bioRe India Ltd. in Madhya Pradesh state (formerly called Maikaal cotton project), where several thousand farmers (2007–2010 between 4’700 and 8’800) produce organic cotton mainly for the European market (www.bioreindia.com). Although the farmers in this cooperative are trained in biodynamic farming, and follow the taught practices to a certain extent, the system is not certified as biodynamic. Nonetheless the products are declared as organic. The farming systems comparison trial presented here was set up in 2007 in Madhya Pradesh state, central India, and is embedded at the training and education center of bioRe Association (www.bioreassociation.org/bioresearch.html). The main aim of the trial is to assess the agronomic, economic and ecological performance of cotton-based farming systems under organic, biodynamic and conventional management including non-Bt and Bt cotton. In this paper, we present the yield and gross margin of cotton, soybean and wheat of the four different farming systems within the first four years after inception of the trial (considered as conversion period in this paper).

## Materials and Methods

### 1 Site description and socioeconomic context

**Figure 1:**
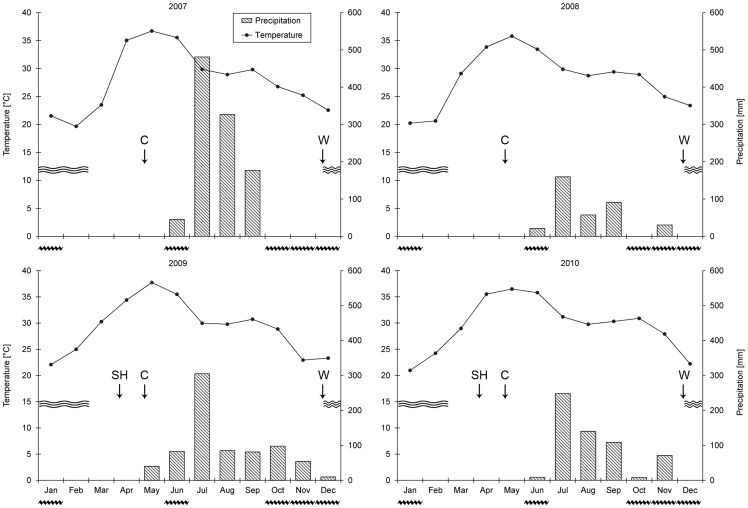
Temperature and precipitation recorded near the trial, Madhya Pradesh, India, 2007–2010, and irrigation practices in the farming systems comparison trial. Vertical arrows (↓) indicate flood irrigation prior to sowing of cotton (C), wheat (W) and sunn hemp (SH). Sunn hemp (green manure) was only grown in 2009 and 2010 on BIODYN and BIOORG plots before cotton sowing. Single closed undulating lines indicate period of drip and flood irrigation in cotton, multiple open undulating lines indicate period of flood irrigation in wheat (wheat received four to five flood irrigations).

The trial site is located in the plains of the Narmada river belt in the Nimar Valley, Khargone district, Madhya Pradesh state, India (22°8′30.3″N, 75°4′49.0″E), at an altitude of 250 meters above sea level. The climate is subtropical (semi-arid), with an average annual precipitation of 800 mm, which occurs in a single peak monsoon season usually lasting from mid-June to September. Temperatures range from 15°C to 49°C with a yearly average of 25°C, and are highest in May/June and lowest in December/January. Climatic data from 2007–2010 obtained near the trial are shown in Figure 1. The trial is located on a fertile Vertisol soil characterized by an average clay content of 600 g kg^−1^ soil, pH (H_2_O) of 8.7, organic C content of 5.0 g kg^−1^ soil, and available P content (Olsen) of 7.0 mg kg^−1^ soil at the start of the trial. Vertisols have shrink-swell characteristics; they cover about 73 million ha of the subtropical (semi-arid) regions of India and are the predominant soil type in Madhya Pradesh [56].

Agriculture is the main livelihood activity in the project area. Farm sizes range from less than 1 ha to more than 10 ha, and soil fertility as well as access to irrigation water vary greatly throughout the region. The major crops in the region are cotton, soybean and wheat. Since 2002, Bt cotton has become very popular and is currently grown on more than 90% of the total area under cotton cultivation in Madhya Pradesh [57], [58]. About 50% of India's organic cotton is produced in Madhya Pradesh [59]. The year consists of three seasons with distinctly different climatic characteristics: The Kharif (monsoon) season is characterized by the monsoon and lasts from June to October. Crops which require humid and warm condition are grown, for example cotton, or soybean. The Rabi (winter) season is characterized by lower temperatures and less rainfall; it lasts from November to March. Crops which require cool temperatures for vegetative growth are grown, for example wheat or chick pea. Finally, the Zaid (summer) season is characterized by hot temperatures and an extensive dry spell; it lasts from March to June. Only farmers with access to irrigation facilities or near river banks grow crops such as melons, gourds or cucumbers in this season. Longer duration crops such as cotton are cultivated during both Kharif and Rabi seasons.

### 2 Trial description

**Figure 2:**
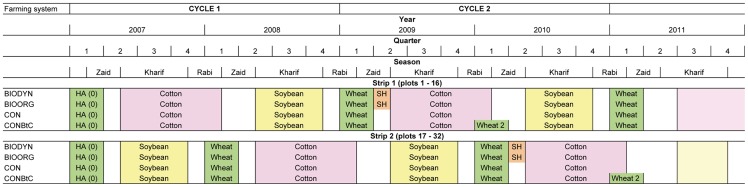
Sequence of crops in different farming systems of the farming systems comparison trial 2007–2010. Seasons: Zaid (summer): March to June, Kharif (monsoon): June to October, Rabi (winter): November to March. HA (0) indicates the homogeneity assessment performed with unfertilized wheat before the implementation of the different farming systems. In 2009 and 2010 Bt cotton was uprooted 2 months earlier to grow a second wheat crop (wheat 2) to reflect common practice of local Bt cotton farmers.

The farming systems comparison trial was established in 2007, and is expected to run for a period of 20 years. Before trial setup, the site was under conventional management by a local farmer. The homogeneity of the terrain was assessed before the implementation of the different farming systems with a test crop of unfertilized wheat (HA (0)) grown from December 2006 to March 2007 (Figure 2). The test crop was harvested using a 5×5 m grid. Data of wheat grain yield, organic C and pH of the soil were used for allocation of strips, blocks and plots (Figure S1).

The trial comprises two organic farming systems (biodynamic (BIODYN), organic (BIOORG)) and two conventional farming systems (conventional (CON), conventional including Bt cotton (CONBtC)). Details of the farming systems are shown in Tables 1 and S1. Organic and biodynamic farming were carried out according to the standards defined by the International Federation of Organic Agriculture Movements (IFOAM) [60] and DEMETER-International [61], respectively. Conventional farming systems followed the recommendations of the Indian Council of Agricultural Research (ICAR) [62] with a slight adjustment to represent local conventional farming systems: farmyard manure (FYM) was applied to account for the integrated nutrient management of local conventional farmers. BIODYN represented the predominant local organic practices, as farmers associated to bioRe India Ltd. (see above) are provided with the respective inputs and trained in biodynamic farming as practiced in the field trial. BIOORG represented general organic practices as practiced in various regions of India where organic cotton is grown (mainly Madhya Pradesh, Maharashtra and Gujarat [59]). CON represented the local conventional practices in Madhya Pradesh before the introduction of Bt cotton in 2002, and CONBtC represented the current local conventional practices.

**Table 1:** Management of the different farming systems compared in a two-year rotation in central India (2007–2010).

Practices	Organic farming systems^1^		Conventional farming systems^2^	
	BIODYN (biodynamic)	BIOORG (Organic)	CON (conventional)	CONBtC (conventional including Bt cotton)
**Genetic material (difference in cotton only)**				
	Non-Bt cotton	Non-Bt cotton	Non-Bt cotton	Bt cotton
**Fertilizer input**				
Type and level (for nutrient inputs see Table S1)	aerobically composted crop residues, weeds, farmyard manure (FYM), and slurry; 19.5-7.7-12.0 t ha^−1^ to cotton-soybean-wheat	aerobically composted crop residues, weeds, farmyard manure (FYM), and slurry; 19.5-7.7-12.0 t ha^−1^ to cotton-soybean-wheat	mineral fertilizers (MOP, SSP, Urea, DAP (wheat only))	mineral fertilizers (MOP, SSP, Urea, DAP (wheat only))
	stacked FYM; 2.8-1.6-2.2 t ha^−1^ to cotton-soybean-wheat	stacked FYM; 2.8-1.6-2.2 t ha^−1^ to cotton-soybean-wheat	stacked FYM; 8.1-3.9-1.6 t ha^−1^ to cotton-soybean-wheat	stacked FYM; 8.1-3.9-1.6 t ha^−1^ to cotton-soybean-wheat
	castor cake; 0.1 t ha^−1^ to cotton (2007 & 2008 only)	castor cake; 0.1 t ha^−1^ to cotton (2007 & 2008 only)		
**Green manure**				
Type and timing of green manure	broadcasted sunn hemp (*Crotalaria juncea*) before cotton in 2009 and 2010 only	broadcasted sunn hemp (*Crotalaria juncea*) before cotton in 2009 and 2010 only	None	None
	hand sown green gram (*Vigna radiata*, 9’070 plants ha^−1^) between cotton rows in all years	hand sown green gram (*Vigna radiata*, 9’070 plants ha^−1^) between cotton rows in all years	None	None
**Plant protection**				
Weed control	bullock-drawn blade or tine harrows	bullock-drawn blade or tine harrows	bullock-drawn blade or tine harrows	bullock-drawn blade or tine harrows
	Hand weeding in cotton	Hand weeding in cotton	Hand weeding in cotton	Hand weeding in cotton
	None	None	Herbicide (2009 and 2010 in soybean and wheat only)	Herbicide (2009 and 2010 in soybean and wheat only)
Insect control and average number of applications per crop rotation (detailed product list, see Table S1)	organic (natural) pesticides 12.5	organic (natural) pesticides 12.25	synthetic pesticides 11.5	synthetic pesticides 11.0
Disease control	None	None	None	None
Special treatments	biodynamic preparations^3^	None	None	None

1 in the text, BIODYN and BIOORG are referred to consistently as organic farming systems, ^2^ in the text, CON and CONBtC are referred to consistently as conventional farming systems, average dry matter content of organic fertilizers: 70%, DAP: Diammonium phosphate, MOP: muriate of potash, SSP: single super phosphate, ^3^biodynamic preparations entailed cow dung (BD-500) and silica powder (BD-501) both stored for six months, and a mixture of cow dung, chicken egg shell powder, basalt rock powder, and plant materials (yarrow, chamomile, stinging nettle, oak bark, dandelion, valerian) stored for 6 months in an open pit (cow pat pit  =  CPP).

The four farming systems mainly differed in the following aspects: Genetic material (cotton only), type and amounts of fertilizer inputs, green manures, plant protection, the use of biodynamic preparations (Table 1, Table S1), and crop sequence (Figure 2). Farming systems are extremely complex, whereby individual management practices are closely linked and interdependent. For instance, it is well known that chemical plant protection is in most cases only economically feasible under conditions of optimal fertilization. That means that we mirror to a certain extent the complexity of a system rather than analyzing effects of single factors, and we intended to mimic common regional practices for the respective farming systems with respect to all management practices, as specified above. This approach is quite common in farming systems research and reflects effects of the system as a whole [20], [21], but does not allow to trace potential differences to individual practices. As a basis for the design of the organic and conventional farming systems served a farm survey of Eyhorn *et al*. [9] in the same region.

The two-year crop rotation consisted of cotton (*Gossypium hirsutum* L.), soybean (*Glycine max* (L.) Merr.) and wheat (*Triticum aestivum* L.) (Figure 2). While in organic farming systems green gram (*Vigna radiata*) was grown between cotton rows in all four years and sunn hemp (*Crotalaria juncea*) was used as a preceding green manure crop for cotton in 2009 and 2010 (crop cycle 2), none of these practices were followed in conventional farming systems. Both green manure crops were cut at flowering and incorporated to the soil. In order to compare the CON and CONBtC farming systems as a whole (rather than the effect of the Bt gene), both the fertilizer dose and crop sequence was adapted (Figure 2).

Fertilizer inputs relied mainly on synthetic products in conventional farming systems (depending on crop between 68 and 96% of total nitrogen (N_total_) applied (Table S1)), whereas organic farming systems received nutrients from organic sources only (Table 1). Organic fertilizers were compost, castor cake, and FYM. Compost was prepared using crop residues, weeds, FYM, and slurry from biogas plants (fed with fresh FYM) as raw materials. FYM was also applied in both conventional farming systems. The relatively high levels of organic fertilizer inputs (Table 1) reflect practices of local smallholder farmers who usually apply some 18.5 t ha^−1^ fresh matter of compost to cotton. On average, compost and FYM contained 0.8-0.6-1.5% and 0.8-0.6-1.6% of N_total_-P_2_O_5_-K_2_O, respectively, whereas castor cake contained 3.3-0.9-0.9% of N_total_-P_2_O_5_-K_2_O. Compost and FYM were broadcasted on the field after land preparation and subsequently incorporated to the soil by bullock-drawn harrows in all farming systems; However, in the organic farming systems in cotton, only 50% of the compost was applied as basal fertilizer input, and the remaining 50% were applied in two equal split applications as top dressings, at square formation and flowering, respectively. Castor cake was applied plant to plant. Nutrient inputs by N-fixing green manure crops were not considered, but will be assessed in future studies (Table S1). Synthetic fertilizers applied in both conventional farming systems were Diammonium phosphate (DAP), Muriate of Potash (MOP), Single Super Phosphate (SSP) and Urea. MOP, SSP and Urea/DAP were applied as basal fertilizer input at sowing time, except in cotton where only 50% of Urea/DAP was applied as basal fertilizer input, and the remaining 50% as a single top dressing at flowering. Across all crops and years, input of N_total_ was 65 kg ha^−1^ in organic farming systems (BIODYN, BIOORG), 105 kg ha^−1^ in CON and 113 kg ha^−1^ in CONBtC (Table S1). The lower inputs of N_total_ in organic compared to conventional farming systems represent local organic practice. The difference in inputs of N_total_ between CON and CONBtC arises from adhering to recommendations by ICAR [62] who advocate systems with Bt cotton to be managed more intensively than systems with non-Bt cotton.

Pest management - including seed treatment - was done with organic (natural) pesticides in organic farming systems, while in conventional farming systems synthetic pesticides were used (Table 1). The type and number of pesticide applications in CON and CONBtC was the same to reflect local farmers' practices [63]. This practice was also confirmed in the survey of Kathage and Qaim [36] comparing conventional Bt and non-Bt cotton in the period 2006–2008 conducted in the four states Maharashtra, Karnataka, Andhra Pradesh, and Tamil Nadu.

The BIODYN system received small amounts of biodynamic preparations (Table 1) consisting of organic ingredients (cow manure, medicinal plants), and mineral compounds (quartz, basalt) which are intended to activate the soil and increase plant health [64]. No significant amounts of nutrients were added by these applications. For further details of biodynamic practices see Carpenter-Boggs *et al*. [64].

With cotton, soybean and wheat the trial represents a cash crop-based farming system in a two-year crop rotation, which is typical for the Nimar Valley in the plains of the Narmada river belt, were the trial is located. Cotton was grown from May to February, except in 2009 and 2010 (crop cycle 2) in CONBtC; in these two years Bt cotton was uprooted two months earlier than in the other three farming systems in order to grow an additional wheat crop (wheat 2) in the Rabi (winter) season (Figure 2). This was done to account for local practices; local conventional farmers noticed that Bt cotton matures earlier than non-Bt cotton, and produces the majority of the yield during the first three months of the harvesting period. Therefore, they started between 2007 and 2010 to grow another wheat crop before the start of the Zaid (summer) season, a practice which was also confirmed by Brookes & Barfoot [65]. Soybean was grown from July to October and followed by wheat from December to March. The land was prepared with bullock-drawn ploughs, harrows and levelers. Cotton was sown by hand at a rate of 0.91 plants m^−2^ (9’070 plants ha^−1^). Soybean and wheat were sown with bullock-drawn seed drills. The inter row and intra row spacing were 30 cm and 4 cm, respectively for both soybean and wheat. In 2007, heavy monsoon rains led to severe waterlogging in the plots which stunted soybean growth and necessitated re-sowing the whole trial. Cultivars were selected according to local practice and availability. In cotton, these were Maruti 9632 (2007), Ankur 651 (2008), Ankur AKKA (2009) and JK Durga (2010) in all farming systems, except in CONBtC where isogenic Bt lines of the same hybrids were used. Non-GM soybean, variety JS-335, and non-GM wheat, variety LOK-1 were cultivated in all farming systems and years. The whole trial was irrigated and all plots received similar amounts of irrigation water; prior to sowing, flood irrigation was carried out on sunn hemp (green manure), cotton and wheat plots (Figure 1). After the monsoon, cotton received additional drip irrigation and two to three flood irrigations to ensure continuous water supply throughout the cropping season. Sunn hemp and wheat received three to four and four to five flood irrigations, respectively. Soybean was grown purely rainfed during the Kharif (monsoon) season. Weeding was done mechanically at 20 (cotton) and 45 (soybean, wheat) days after sowing, using bullock-drawn blade or tine harrows in all farming systems. In cotton, additional hand weeding was carried out. No hand weeding was carried out in soybean and wheat. No synthetic herbicides were applied in conventional farming systems except in soybean and wheat in 2009 and 2010, which reflects the situation of most smallholder cotton farmers in India [66]. Cotton was harvested by several manual hand pickings. Soybean and wheat were harvested manually with sickles, and bound to bundles which were removed from the field and subsequently threshed with a threshing machine.

In order to obtain data from each crop during each year, the layout was doubled with shifted crop rotation in two strips, resulting in a total of 32 plots, and 16 plots per strip (Figure S1). Each farming system was replicated four times in a randomized block design in each of the two strips. Plots are sized 16 m×16 m ( =  gross plot) and time measurements of activities were recorded for gross plots. The outermost 2 m of each plot served as a border, and yield data were only obtained in the inner sampling plot sized 12 m×12 m ( =  net plot) in order to avoid border effects. The distance between two plots within a strip and between the two strips is 6 m and 2 m, respectively. Data was obtained from 2007 to 2010. Data from 2007–2008 belongs to the complete crop rotation of the 1^st^ crop cycle (cycle 1), and data from 2009–2010 to the 2^nd^ crop cycle (cycle 2).

As Bt cotton was commercially released in India in 2002, no official approval of the study was required. The land needed for the farming systems comparison trial was purchased and belongs to bioRe Association. No protected species were sampled.

### 3 Data consolidation and economic calculations

Calculations of gross margins required consolidation of production costs. We only considered variable (operational) production costs in our study, excluding interest rates for credits. We included input costs, labor costs for field activities (including e.g. compost preparation), and costs associated with the purchase of inputs from the local market. Time measurements on gross plots and farmers' fields were complemented with data obtained in expert meetings with experienced farmers and local extension officers. Variable production costs for cotton (Table S2), soybean (Table S3), and wheat (Table S4) were cross-checked with the values reported by the Ministry of Agriculture, Government of India [67]. Gross margins were obtained by subtracting the variable production costs from the gross return ( =  yield * price per unit). Prices (products, inputs, labor) corresponded to local market conditions and were adapted each year (Table 2, Table S2). A premium price for organic cotton was considered in 2010 only (after three years conversion period according to IFOAM standards).

**Table 2:** Domestic market prices of cotton, soybean and wheat, premium prices on organic cotton and prices per working hour 2007–2010 in Khargone district, Madhya Pradesh, India.

Year	Commodity				
	Cotton [INR kg^−1^]	Cotton premium price [INR kg^−1^]	Soybean [INR kg^−1^]	Wheat [INR kg^−1^]	Labor [INR h^−1^]
2007	23.3	4.7 (n.c.)	15.5	10.4	7.5
2008	26.8	3.3 (n.c.)	20.0	11.0	9.0
2009	31.5	3.3 (n.c.)	22.5	12.0	11.3
2010	49.0	4.0 (c.)	22.5	12.0	12.5

n.c.: not considered in economic calculations (conversion  =  first three years, according to IFOAM standards), c.: considered in economic calculations; No premium exists for organic soybean and wheat due to local market structures; Exchange rate INR: USD  =  50∶1 (source: http://eands.dacnet.nic.in/AWIS.htm, stand October 2012).

### 4 Statistical analysis

Data exploration revealed four outliers which were removed from the dataset. The reason was heavy monsoon rains and subsequent water-logging in four plots in 2009 (Plots 11 and 27 (both BIOORG), and plots 12 and 28 (both BIODYN), Figure S1).

Yield and gross margin data of each crop, and of the complete crop rotation (cotton+wheat 2+soybean+wheat) were analyzed separately with linear mixed effect models using the function lme from the package nlme [68] of the statistical software R version 2.15.2 [69]. We checked our data for model assumptions graphically (normal Q-Q of fixed and random effects, Tukey-Anscombe and Jitter plots) and no violation was encountered. We used a model with *System*, *Cycle*, the interaction of *System*×*Cycle* and *Strip* as fixed effects, and *Year* (n = 4), *Block* (n = 4) and *Pair* (n = 16) as random intercepts.

The fixed effect *Cycle* was included in the model to account for repeated measures on the same plot (e.g. cotton on plot 1 in 2007 and in 2009) and allows a partial separation of *Cycle* and *Year* effects due to the shifted crop rotation in the two strips as proposed by Loughin [70] for long-term field trials. *Cycle* effects give an indication how the situation changes across the timeframe of the trial. However, as we only have two levels of *Cycle* (thus Df = 1 for *Cycle* in the ANOVA) at this stage of the trial, we have little statistical power to detect *Cycle* effects. The same applies to the fixed effect *Strip*. We nevertheless included *Cycle* and *Strip* into our model to separate the *System* effect from possible *Cycle* and *Strip* effects. To account for similar conditions of neighboring plots (e.g. Plots 1 and 17, 2 and 18, etc., Figure S1) we included the random intercept *Pair* with 16 levels.

For yield and gross margin data of the complete crop rotation, the random intercept *Year* was removed from the model, as data from two years were compiled. Significant *System*×*Cycle* interactions suggested that the main effects of *System* and *Cycle* have to be interpreted with caution; As the effects of the different systems were not consistent across cycles, we split the datasets and performed post-hoc multiple comparisons for the fixed effect *System* separately for each cycle (method: Tukey, superscript letters after cycle-wise values in Tables 3 and 4). In the case of gross margin data of soybean, no significant *System*×*Cycle* interaction was encountered. Therefore, we performed post-hoc multiple comparisons on the whole dataset of cycle 1 and cycle 2 together (superscript letters after average values in Table 4). We defined a difference to be significant if *P* <0.05 (α = 0.05).

**Table 3:** Mean yields [kg ha^−1^] of cotton, soybean and wheat, and total productivity per cycle and across four years (2007–2010) in the farming systems compared in central India.

Farming system	Crop								Total productivity of crop rotation	
	Seed cotton	SEM	Wheat 2 grains	SEM	Soybean grains	SEM	Wheat grains	SEM	Seed cotton + Wheat 2 grains + Soybean grains + Wheat grains	SEM
**Cycle 1 (2007–2008)**										
BIODYN	2'047^ c^	68	-	-	1'399^ a^	158	2'997^ c^	153	6'443^ b^	104
BIOORG	2'072^ c^	49	-	-	1'536^ a^	192	2'831^ c^	121	6'440^ b^	187
CON	2'700^ b^	141	-	-	1'483^ a^	155	4'262^ a^	221	8'444^ a^	146
CONBtC	3'133^ a^	176	-	-	1'473^ a^	195	3'730^ b^	272	8'336^ a^	254
**Cycle 2 (2009–2010)**										
BIODYN	1'894^ a^	108	-	-	1'807^ ab^	87	3'338^ a^	207	7'039^ b^	268
BIOORG	1'942^ a^	103	-	-	1'739^ b^	117	3'303^ a^	191	6'984^ b^	239
CON	1'614^ a^	43	-	-	1'993^ a^	108	3'273^ a^	175	6'880^ b^	119
CONBtC*	1'834^ (a)^	179	1'573	169	1'997^ a^	161	3'481^ a^	182	8'885^ a^	390
**Average (2007–2010)**										
BIODYN	1'971	64	-	-	1'603	104	3'167	132	6'741	270
BIOORG	2'007	56	-	-	1'638	114	3'067	125	6'712	257
CON	2'157	157	-	-	1'738	113	3'767	187	7'662	455
CONBtC	2'484	207	(787)	-	1'735	140	3'605	161	8'610	376
**ANOVAs of linear mixed effect models**										
Source of variation	*P* value	Df	-	-	*P* value	Df	*P* value	Df	*P* value	Df
System (S)	<0.001	3	-	-	0.102	3	<0.001	3	<0.001	3
Cycle (C)	<0.001	1	-	-	0.066	1	0.686	1	0.912	1
Strip	0.141	1	-	-	0.472	1	0.960	1	0.002	1
S×C	<0.001	3	-	-	0.039	3	<0.001	3	<0.001	3

SEM: standard error of the mean, BIODYN: biodynamic, BIOORG: organic, CON: conventional, CONBtC: conventional with Bt cotton, different superscript letters indicate significant difference between farming systems within one Cycle (Tukey test, *P*<0.05), * in 2009 and 2010 Bt cotton was uprooted 2 months earlier to grow a second wheat crop (wheat 2) to reflect common practice of local Bt cotton farmers (for the sequence of crops in different farming systems see Figure 2), *P* value and degrees of freedom (Df) of fixed effects in linear mixed effect models, random factors in the model: Year (n = 4), Block (n = 4), Pair (n = 16), for total productivity random factor Year was excluded as data from two years were compiled.

**Table 4:** Mean gross margins [INR ha^−1^] of cotton, soybean and wheat, and total gross margin per cycle and across four years (2007–2010) in the farming systems compared in central India.

Farming system	Crop								Total gross margin of crop rotation	
	Seed cotton	SEM	Wheat 2 grains	SEM	Soybean grains	SEM	Wheat grains	SEM	Seed cotton + Wheat 2 grains + Soybean grains + Wheat grains	SEM
**Cycle 1 (2007–2008)**										
BIODYN	38'243^ c^	2'226	-	-	19'211	4'210	26'044^ c^	1'584	83'498^ b^	8'021
BIOORG	38'676^ c^	1'203	-	-	21'830	4'858	24'420^ c^	1'291	84'926^ b^	8'268
CON	51'792^ b^	2'779	-	-	18'401	4'093	37'099^ a^	2'221	107'292^ a^	4'546
CONBtC	60'811^ a^	4'851	-	-	18'147	4'719	31'361^ b^	2'832	110'319^ a^	8'308
**Cycle 2 (2009–2010)**										
BIODYN	62'786^ a^	7'217	-	-	32'176	1'683	30'764^ a^	2'374	125'726^ a^	6'721
BIOORG	64'490^ a^	6'714	-	-	30'812	2'278	30'443^ a^	2'181	125'745^ a^	9'354
CON	42'962^ b^	4'653	-	-	28'949	3'724	24'773^ b^	2'028	96'683^ b^	6'701
CONBtC*	43'810^ (b)^	2'995	4'837	2'190	29'399	4'644	27'037^ b^	2'117	105'082^ b^	5'232
**Average (2007–2010)**										
BIODYN	50'514	4’918	-	-	25'694^ ab^	2’758	28'404	1’507	104'612	7'461
BIOORG	51'583	4’789	-	-	26'321^ a^	2’865	27'432	1’450	105'335	7'008
CON	47'377	2’852	-	-	23'675^ b^	2’780	30'936	2’155	101'988	3'089
CONBtC	52'310	3’165	(2'418)	-	23'773^ b^	3’311	29'199	1’797	107'701	3'849
**ANOVAs of linear mixed effect models**										
Source of variation	*P* value	Df	-	-	*P* value	Df	*P* value	Df	*P* value	Df
System (S)	0.115	3	-	-	0.006	3	0.022	3	0.298	3
Cycle (C)	0.046	1	-	-	0.158	1	0.606	1	<0.001	1
Strip	0.001	1	-	-	0.469	1	0.805	1	<0.001	1
S×C	<0.001	3	-	-	0.150	3	<0.001	3	<0.001	3

SEM: standard error of the mean, BIODYN: biodynamic, BIOORG: organic, CON: conventional, CONBtC: conventional with Bt cotton, different superscript letters indicate significant difference between farming systems within one Cycle (Tukey test, *P*<0.05), * in 2009 and 2010 Bt cotton was uprooted 2 months earlier to grow a second wheat crop (wheat 2) to reflect common practice of local Bt cotton farmers (for the sequence of crops in different farming systems see Figure 2), *P* value and degrees of freedom (Df) of fixed effects in linear mixed effect models, random factors in the model: Year (n = 4), Block (n = 4), Pair (n = 16), for total gross margin random factor Year was excluded as data from two years were compiled.

## Results and Discussion

### 1 Yield

**Figure 3:**
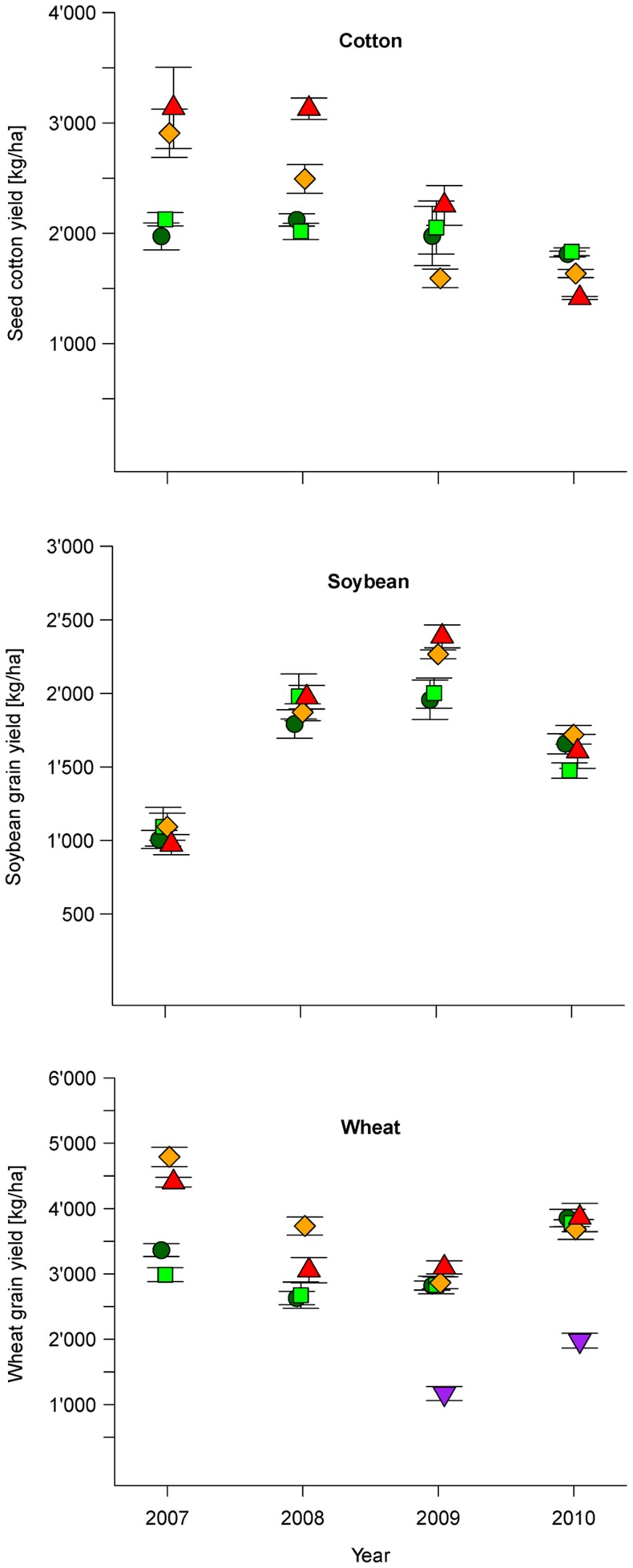
Yield (mean ± standard error) 2007-2010 in cotton, soybean and wheat. Farming systems: (•) biodynamic (BIODYN), (▪) organic (BIOORG), (♦) conventional (CON), (▴) conventional with Bt cotton (CONBtC), (▾) wheat after Bt cotton (wheat 2); In 2009 and 2010 Bt cotton was uprooted 2 months earlier to grow a second wheat crop (wheat 2) to reflect common practice of local Bt cotton farmers. Non-GM soybean and wheat varieties were cultivated in the CON and CON-BtC plots throughout the trial. Note the different scales on y-axes in the different panels of the graph.

Cotton yields (seed cotton, picked bolls containing seed and fiber) were, averaged across the four years, 14% lower in organic (BIOORG, BIODYN) compared to conventional farming systems (CON, CONBtC). This is in the same range as the findings of a study conducted in Kyrgyzstan [27]. The *System*×*Cycle* interaction had a significant effect (*P*<0.001) on cotton yields (Table 3). The difference in yield was very pronounced in cycle 1 (2007–2008, +42% yield increase in conventional farming systems), while yields were similar among all farming systems in cycle 2 (2009–2010) (Figure 3). CONBtC consistently showed higher yields than the three other farming systems, except in 2010. This is in line with the findings of several international meta-studies, which also reported generally higher yields and increased profitability in Bt cotton compared to non-Bt cotton production [34], [65], [71]. However, cotton yield increases through the use of Bt seeds may vary greatly among regions (from zero in Australia, up to 30% in Argentina) due to e.g. varieties used in Bt and non-Bt production, and effectiveness of chemical plant protection in non-Bt production [65]. Glover [72] also points out that the performance and impacts of Bt crops have been highly variable, socio-economically differentiated and contingent on a range of agronomic, socio-economic and institutional factors, thus underlining that the contextual interpretation of results is of paramount importance. The cotton yields per hectare of CONBtC in cycle 1 in our study were in the same range reported by Konduru *et al*. [73]. The severe decline in yield observed for CONBtC in cycle 2 when compared to cycle 1 (Figure 3) can be partly explained by the fact that Bt cotton plants were uprooted two months earlier than plants in other farming systems in cycle 2 (see chapter 2.2). However, this does not explain the decline in yield observed from 2007 to 2010 in the CON farming system, in which cotton plants were not uprooted. In cycle 1 the cotton yields in CONBtC were 16% higher than in CON (Table 3) which could be due to both the effect of the Bt gene products on pests (as isogenic hybrids were used) as well as the higher input of fertilizer (166 kg N ha^−1^ *vs*. 146 kg N ha^−1^) recommended for Bt cotton. The difference in yield between Bt and non-Bt cotton in our study was much smaller than the differences in yield reported by others for India [33], [36], [38]–[40], [74], indicating that the chemical plant protection applied to the CON system in our experiment was relatively effective. In contrast to the conventional systems, both of the organic farming systems showed rather stable cotton yield throughout the entire experimental period (Figure 3). As cycle effects and the *System*×*Cycle* interaction are confounded by year effects, we have to consider that in 2009 and 2010 the cotton yield was generally lower than in 2007 and 2008, as was confirmed by statistical yield data of the state Madhya Pradesh [67]. Apparently, the conventional farming systems could not realize their yield potential due to the less advantageous growing conditions in cycle 2 (rainfall and water logging in the harvest period October – December, Figure 1). The organic farming systems however, were not affected by the disadvantageous conditions in cycle 2 (Figure 3). An additional nitrogen fixing green manure pre-crop, planted before cotton in the organic systems in cycle 2 (Figure 2), may have contributed to the observed stability in yield in these systems through the consistent provision of nitrogen to the plants. Cotton yields of future crop cycles will thus determine whether productivity in conventional systems will reach their initial high level as well as determine whether yields of organic farming systems will start to increase. A yield depression is usually observed during the conversion to organic farming in India [75]. However, in our trial no such trend was oberserved between cycle 1 and cycle 2.

Non-GM soybean and wheat varieties were cultivated in both CON and CONBtC systems. In 2007, average soybean yields across all farming systems were 45% lower compared to the other three years (Figure 3), as the whole trial had to be re-sown due to severe water logging. Soybean yields were, averaged across the four years, 7% lower in organic compared to conventional farming systems. The *System*×*Cycle* interaction had a significant effect (*P*<0.05) on soybean yields (Table 3). No significant difference in yield could be identified between farming systems in cycle 1. However, in cycle 2 CON and CONBtC showed significantly higher yields than BIOORG (Table 3). This is likely due to higher pest incidences and thus lower yields in organic systems in 2009. BIODYN produced similar soybean yields as both conventional systems throughout the experimental period (*P*>0.05). The 1% and 11% lower yields in organic farming systems in cycle 1 and 2, respectively, are considerably lower than the 18% lower yields reported for organic soybean in the Karnataka region [76]. These results indicate similar productivity of conventional and organic soybean production systems under subtropical (semi-arid) conditions and suggest that in similar settings no further yield gains can be achieved through the provision of synthetic inputs compared to organic management practices. The smaller difference in yield between conventional and organic soybean - when compared to cotton and wheat (see below) - could be explained by considering the plant type. Soybean is the only legume crop in the crop rotation, possessing the ability to fix atmospheric N, thereby avoiding potential nitrogen shortage for optimal plant growth. These results confirm the findings of Seufert *et al*. [17] whose meta-analysis showed a lower yield gap between conventional and organic legume crops when compared to non-legume crops, and indicate that cotton and wheat yields in organic farming systems in our trial may be restricted by nitrogen limitation in the soil.

Wheat yields were, averaged across the four years, 15% lower in organic compared to conventional farming systems, which is similar to the 20% yield gap reported for Uttarakhand [76]. The *System*×*Cycle* interaction had a significant effect (*P*<0.001) on wheat grain yield. Similar to cotton, there was a significant yield gap between conventional and organic farming systems in cycle 1 (+37% yield increase in conventional farming systems), but not in cycle 2 due to both slightly lower yields in the conventional systems and slightly higher yields in the organic systems compared to cycle 1 (Table 3, Figure 3). For both soybean and wheat no yield differences were detected between CON and CONBtC farming systems, except for significantly higher wheat yields in CON in cycle 1 (Table 3).

Regarding the total productivity per crop rotation in terms of summed-up dry matter yields of cotton, soybean and wheat (including wheat 2 in CONBtC in cycle 2), a significant effect (*P*<0.001) of the *System*×*Cycle* interaction was found (Table 3). Both of the conventional farming systems were significantly more productive (+30%, Table 3) than the organic farming systems in cycle 1. However, in cycle 2 only CONBtC showed significantly higher productivity (+28%, Table 3) when compared to the other three farming systems, due to the additional wheat crop (wheat 2). Differences in yield between BIODYN and BIOORG were minor and not statistically significant for all crops and total productivity of the whole crop rotation (Table 3). Unexpectedly, there was a significant *Strip* effect for total productivity across the whole crop rotation. This may be explained by the fact that different crops were cultivated on the two strips in a given year (Figure 2). The compilation of whole crop rotations (compiling years) thus led to the combination of the observed variability for each crop across the four years, and subsequently to the *Strip* effect becoming significant (Table 3).

In general, the first four years of the farming systems comparison trial in India revealed that there was a significant yield gap in cycle 1 (2007–2008) for cotton (−29%) and wheat (−27%), in organic compared to conventional farming systems, whereas in cycle 2 yields of the three crops were similar in all farming systems due to low yields in the conventional systems (Table 3). Because there was no clear trend of yield development for cotton and wheat during the four year period in any of the systems, observed results rather reflect growth conditions in respective years than long-term yield trends of cotton and wheat. However, the marginal yield gap between the BIOORG system and the conventional systems, and the par soybean yields of BIODYN and the conventional systems show that leguminous crops are a promising option for conversion to organic systems under the given conditions. The yield development across the whole crop rotation needs to be verified during future crop cycles.

### 2 Economic analysis

The production costs (i.e. labor and input costs) in our trial (Tables S2, S3 and S4) were in a similar range as reported by the Ministry of Agriculture, Government of India [67]. The variable production costs of conventional (CON, CONBtC) compared to organic (BIOORG, BIODYN) farming systems were on average 38%, 66%, and 49% higher in cotton, soybean and wheat (Table S2, S3 and S4). This is in agreement with findings for cotton in Gujarat, but contradicts findings for wheat in Punjab and Uttar Pradesh [53]. The main reason for the differences observed in our study were the higher input costs (fertilizer, pesticides) in the conventional farming systems, which is in accordance with the findings of a study conducted in Kyrgyzstan [27]. Labor costs were similar among all farming systems, as organic and conventional farming systems did not differ greatly with regard to time requirements of activities (Table S2, S3 and S4). For instance, weeding was done manually in all systems and no herbicides were applied in the conventional farming systems except for soybean and wheat in cycle 2, reflecting the common practice of most smallholder cotton farmers in India [66]. This practice, however, might change in the near future, as labor costs in Indian agriculture are on the rise [77]. The variable production costs of the two organic farming systems were similar for all crops. This was also true for the two conventional farming systems, except for cotton, where the variable production costs of CONBtC were 17% higher compared to CON due to both the higher seed price of Bt cotton (Table S2) [34], [36], [47] and production cost of the additional wheat crop in cycle 2. The prices we present here for Bt seed material are in the same range as reported by Singh *et al*. [78].

Cotton was the most important cash crop and accounted for 48% of the total gross return in the crop rotation, irrespective of the system. The *System*×*Cycle* interaction had a significant effect (*P*<0.001) on cotton gross margins (Table 4). Due to much higher yields, conventional cotton led to 32% higher gross margins compared to organic cotton in cycle 1 (2007–2008), which is in accordance with several international meta-studies [34], [65], [71]. However, the opposite was true in cycle 2 (2009–2010), where we observed 32% lower gross margins in conventional cotton, supporting the findings of Bachmann [27]. The significant *Strip* effect for cotton gross margin can be explained by the highly variable cotton prices across the four years (Table 2).

For soybean, the *System*×*Cycle* interaction was not significant (Table 4) which allowed for an analysis of gross margin data across both cycles (see 2.4). Considerably higher gross margins were obtained in organic systems (+10%) compared to conventional systems between 2007 and 2010. The difference was statically significant for BIOORG (+11%, *P*<0.05) and almost significant for BIODYN (+8%, *P*<0.1). These results indicate that the slightly lower productivity of organic soybean was balanced out by lower production costs rendering soybean production considerably more profitable in organic systems when compared to conventional farming systems.

For wheat gross margins, the *System*×*Cycle* interaction was found to be significant (*P*<0.001, Table 4). Under organic farming, wheat obtained significantly lower gross margins in cycle 1 (−26%), but significantly higher gross margins (+18%) in cycle 2 (Table 4). The earlier removal of Bt cotton from the field in order to grow another wheat crop in CONBtC, before the start of the Zaid (summer) season in cycle 2, only provided minor economic benefits compared to CON (Table 4). This was mainly due to low yields of wheat 2 (<50% compared to regular wheat crop, Figure 3) and lower market prices for wheat compared to cotton (Table 2). Thus, the additional wheat crop could not compensate for the missed cotton yield of the last picking period with respect to economic profitability, a result contradicting total yield performance across all crops.

**Figure 4:**
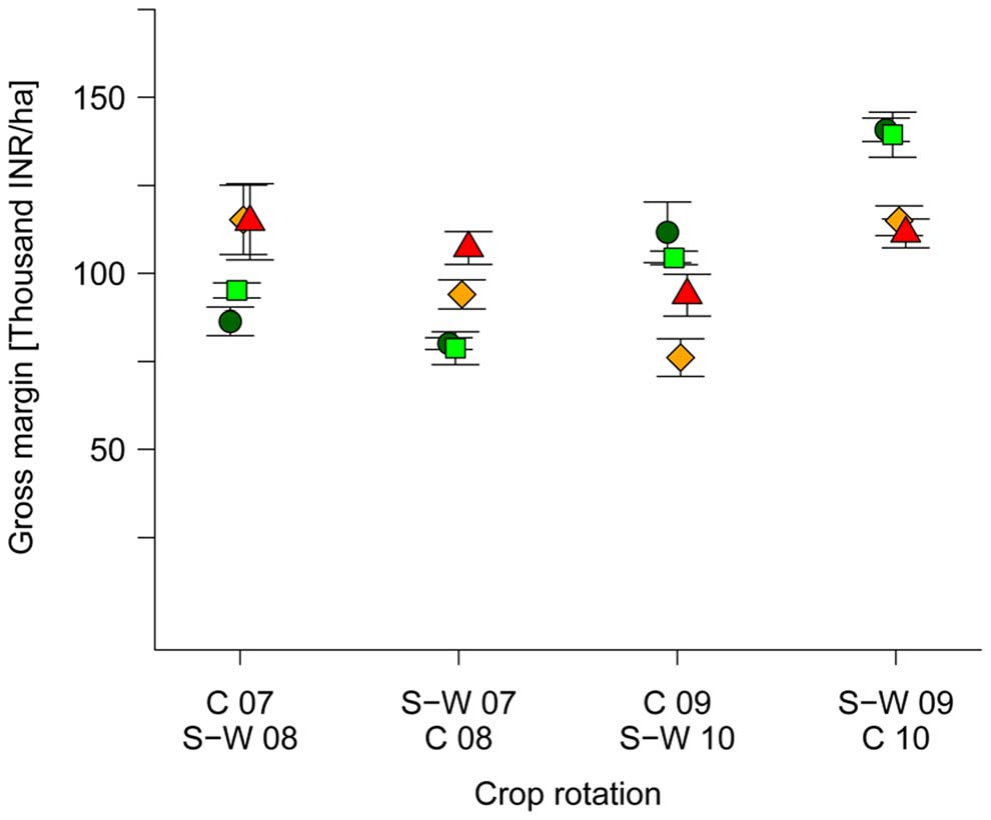
Gross margins (mean ± standard error) of four crop rotations. Farming systems: (•) biodynamic (BIODYN), (▪) organic (BIOORG), (♦) conventional (CON), (▴) conventional with Bt cotton (CONBtC) (includes wheat cultivated after Bt cotton on the same plots in 2009 and 2010); C =  cotton, S-W =  soybean-wheat; Exchange rate Indian rupee (INR): US Dollar (USD)  = 50∶1 (stand October 2012), premium price on organic cotton only in 2010.

A highly significant (P<0.001) *System*×*Cycle* interaction was found for the total gross margin per crop rotation. In cycle 1, favorable weather conditions allowed for the realization of the anticipated yield potential in conventional farming systems, and thus led to both higher cotton and wheat yields (Figure 3), and concomitantly significantly higher gross margins (+29%, Table 4). However, In cycle 2 the gross margins of the organic farming systems were significantly higher (+25%) (Table 4, Figure 4) due to par yields as measured in the conventional systems (Figure 3), but lower variable production costs (Tables S2 and S4). If the premium price in 2010 would not have been considered, the total gross margin per crop rotation in cycle 2 would not be substantially lower and still be significantly higher in organic farming systems (statistical analysis not shown). This could imply that, in favorable years (e.g. good yield, high price for commodities, etc.), premium prices are not required for achieving comparable economic returns in organic and conventional farming systems. However, the premium is needed in unfavorable years in order to compensate for yield gaps, and to avoid that organic farmers sell their produce to the local conventional market. The significant *Strip* effect for total gross margin can be explained by compiling the individual gross margins of the different crops, thereby transferring the significant *Strip* effect of cotton to the total gross margin (Table 4).

The results of cycle 2 suggest that under certain conditions, organic farming can be an economically viable and less capital-intensive production system compared to conventional farming systems, which is in accordance with the findings by Ramesh *et al*. [76] and Panneerselvam *et al*. [79]. However, long-term studies are needed in order to substantiate these findings. Moreover, the viability of organic farming systems strongly depends on farmers having access to knowledge, purchased inputs such as organic fertilizers, pesticides and non-GM seeds, and assuming that there is a market demand and well developed certification system. These are vital components for the economic profitability of organic farming systems [27] especially against the backdrop of increasing labor costs in Indian agriculture [77]. The costs for organic certification are substantial in case individual farmers have to undergo this process, and premium prices may also have to cover these costs. Up to now, certification costs are usually covered by the cotton organization that is purchasing seed cotton from smallholders (here: bioRe India Ltd.). This includes extensive testing of seeds and seed cotton for GM contamination, as well as the implementation of Tracenet, an internet based electronic service offered by the Agricultural and Processed Food Products Export Development Authority (APEDA) for facilitating certification of organic export products from India which comply with the standards of the (National) Programme for Organic Production (NPOP/NOP). This is a big challenge of certified organic cotton compared to fair trade cotton [15], and further organic cotton initiatives rely on cost-efficient and trustful certification and education programs as well.

### 3 Transferability of field trial results

So far little is known about the comparative performance of cotton-based farming systems under organic and conventional management. To our knowledge, this is the first publication comparing the agronomic and economic performance of biodynamic, organic, conventional and conventional with Bt cotton-based farming systems. The few studies published to date compared either organic *vs*. conventional [9], [28], [80] or conventional *vs*. conventional with Bt cotton production systems [36].

By including two organic (BIOORG and BIODYN) and two conventional (CON and CONBtC) farming systems in our trial, we were able to cover a wide range of current cotton-based farming systems in central India (see 2.2). Forming close links to local partners and having practitioners in the steering committee of our systems comparison trial guaranteed that the various agronomic activities that were involved represented local farmers' practice. Due to the cooperative initiative of bioRe, cotton farmers are trained in compost preparations, and organic inputs are purchased collectively and distributed among the farmers. Farmers associated with bioRe may not face the various problems commonly observed during conversion to organic farming [75] to a similar extent as do farmers without affiliation to similar institutions. This is likely due to the experienced and well-functioning extension service of bioRe. Drip irrigation is strongly promoted and subsidized by the Indian government and is not specific to our experiment. However, a direct extrapolation of our results to the reality of smallholder farmers is not possible due to the fact that farmers are confronted with several obstacles not considered in our study; These are for example market access, access to inputs and know-how and in particular costs associated with the organic certification process (see 3.2). One also has to consider that yield estimates from optimally managed trial plots are usually higher than the average yield of smallholder farmers. This is due to the fact, that such optimal crop management, as it was applied in this farming systems comparison trial, might not always be possible under the real-world smallholder conditions. This is especially true as the trial was conducted on a fertile Vertisol soil. Based on a survey of more than 1’000 smallholders in Madhya Pradesh, the average yield levels in the respective time period (2007–2009) were 1’416 kg ha^−1^, 1’285 kg ha^−1^, and 2’426 kg ha^−1^ for seed cotton, soybean, and wheat [67], as compared to 2’585 kg ha^−1^, 1’761 kg ha^−1^, and 3’658 kg ha^−1^ found in our trial. In a survey performed between 2006 and 2008 among 700 smallholder cotton farmers in India, average yields of 1’743 and 2’048 kg ha^−1^ seed cotton were reported for conventional (without Bt) and Bt cotton, respectively [36], as compared to 2’700 kg ha^−1^ and 3’133 kg ha^−1^ in our trial in the same time period (2007–2008). Thus, our yields might be generally overestimated, but within the range of other field trials in India [78].

The following examples show that comparative findings on yield and economics between organic and conventional cotton are highly contextual. Eyhorn *et al*. [9] surveyed more than 50 conventional (without Bt cotton) and 30 organic cotton farmers in the Nimar Valley, Madhya Pradesh, India, during the period of 2003–2004. Their findings support our results of cycle 2 (years three and four, 2009–2010): yields of cotton and other cash crops were on par with conventional farmers, but with economic benefits for the organic farmers due to lower production costs. These findings also underline the practical relevance of our results for cotton production in the in the smallholder context in Madhya Pradesh. Likewise, Venugopalan *et al*. [81] reported similar or slightly higher cotton yields in an organic compared to a non-organic system under low input and semi-arid conditions in the Yavatmal district, Maharashtra, India (observation phase 2001–2005). In a long-term trial under rainfed conditions in Nagpur (Maharashtra, India), Menon [82] reported a yield gap of 25% of organic cotton compared to the modern method of cultivation ( =  conventional without Bt cotton) within the first six years after conversion (1994–2000). Thereafter (2002–2004), the organic farming systems outyielded the conventional systems by up to 227 kg seed cotton ha^−1^ [28].

However, our findings from India are in contrast to the results from a survey on cotton farms in Northern San Joaquin Valley, California [80]. There, averaged over a six-year observation period, yields of organic cotton were 19 and 34% lower (*P*<0.05) than those of cotton under conventional and integrated pest management (reduced insecticide input). It has to be taken into account that for two out of the six years assessed in their study, different varieties were compared under conventional and organic management. Production costs of organic cotton were 25 and 60% higher than those of cotton under conventional and integrated pest management, respectively. This was mainly due to the higher labor costs for manual weeding. In our trial, there was less difference in weed control, as manual weeding is still the common practice of most smallholder cotton farmers in India [66]. This example underlines the contextual nature of the findings concerning the agronomic and economic performance between organic and conventional cotton, which was also pointed out by Seufert *et al*. [17] and de Ponti *et al*. [16] for other crops than cotton.

Building on unique panel data on Indian cotton farming of smallholder farmers between 2002 and 2008, Kathage and Qaim [36] showed that the use of conventional Bt cotton led to a 24% yield increase and a 50% gain in cotton profit compared to smallholders growing conventional non-Bt cotton. In contrast to the systematic farm survey by Kathage and Qaim [36], our study represents a pairwise comparison of cotton-based farming systems under identical pedo-climatic conditions. While the study by Kathage and Qaim [36] can better depict the actual situation on real farms for a given region, our results can better represent the potential outcomes that are achievable under optimal conditions with respect to inputs and knowledge access. In our study, there were comparatively little differences between the CONBtC and the CON farming systems regarding cotton yield (+16 and +14% in cycle 1 and 2, respectively) and cotton gross margin (+17 and +2% in cycle 1 and 2, respectively). Furthermore, it needs to be taken into account that with the introduction of Bt cotton to India in 2002, the provincial governments began to subsidize Bt cotton considerably, especially between the years of 2002 and 2008. This led to the rapid spread of Bt cotton and the breakdown of the non-Bt cotton seed chain. The relatively weak performance of the non-Bt cotton in the farm survey by Kathage and Qaim [36] could partly be explained by the poor quality of non-Bt cotton seeds, as propagation of non-Bt cotton was abandoned and led to limited availability of non-Bt cotton seeds from old stocks of probably poor quality and mainly older cultivars [59], [83].

In contrast to Kathage and Qaim [36], our study also includes other cash crops such as soybean and wheat as part of cotton-based farming systems. These are essential components for enabling long-term cotton cropping and for securing livelihoods of smallholders, as they enable the distribution of risks. According to our findings, the investigated organic farming systems also showed a significant yield gap compared to the conventional farming systems in wheat in cycle 1, as well as for total productivity per crop rotation (including additional wheat crop after Bt cotton (wheat 2)) in cycle 2. Furthermore, a smaller yet significant yield gap was observed for soybean in cycle 2 for the BIOORG system (but not for the BIODYN system) (Table 3). Nevertheless, as in our study organic farming systems were less capital-intensive than conventional ones for all crops, they may be of particular interest to smallholder farmers who often do not have the financial means to purchase inputs and would thus need to seek loans. If this can be verified in on-farm trials, organic farmers might be less exposed to financial risks associated with fluctuating market prices of synthetic fertilizers and crop protection products [27], [79]. Additional on-farm investigations have been started in order to classify regional farms into several farm typologies with corresponding levels of available production factors. This should allow for the assessment of the perspectives of each farm type regarding conversion to organic farming systems. If organic farming is to be adopted more widely, more inter- and transdisciplinary research giving focus to the problems and benefits of organic management practices needs to be undertaken [84]. Furthermore, large efforts have to be made to gather and disseminate knowledge on production techniques. Intensifying research on organic farming systems to a similar extent as was the case for research on GM crops [85] may help to provide additional relevant information to policy makers, advisors and farmers about comparative advantages and limitations of different cotton production systems.

## Conclusions

With this publication we respond to the urgent need for farming systems comparison trials in the tropics and subtropics [17], [26]. Here we show results from the conversion period (first four years after inception of the trial) of cotton-based farming systems representative for Vertisol soils in Madhya Pradesh, central India. Due to the short-term nature of our results and the observed *System*×*Cycle* interactions (no clear trend of system performance over time) for yield and gross margin data of cotton and wheat, definitive conclusions about the comparative agronomic and economic performance of the investigated farming systems cannot be drawn. However, our results show that organic soybean productivity can be similarly high as in conventional systems at lower input levels, which can make organic soybean production - as part of cotton-based crop rotations - more profitable. Future research will bring further insights on agronomic and economic performance of the different farming systems after the conversion period, thus providing indications on the long-term sustainability across the whole crop rotation. Furthermore, the effects of the farming systems on biodiversity, soil fertility, other ecological co-benefits such as climate change mitigation by means of C sequestration, and product quality need to be elucidated.

## Supporting Information

Figure S1 **Experimental design of the farming systems comparison trial in Madhya Pradesh, India.** Farming systems: biodynamic (BIODYN), organic (BIOORG), conventional (CON), conventional with Bt cotton (CONBtC), CONBtC includes wheat cultivated after Bt cotton on the same plots in 2009 and 2010, open squares belong to Strip 1, closed squares belong to Strip 2, distance between two plots within a strip  = 6 m, distance between the two strips  = 2 m.
(TIF)

Table S1 Fertilizer and plant protection practices in the farming systems compared in central India (2007–2010). BIODYN: biodynamic, BIOORG: organic, CON: conventional, CONBtC: conventional with Bt cotton, Ntotal: total nitrogen, OF: organic fertilizers (compost, FYM and castor cake), Ntotal includes only fertilizer derived N, nutrient inputs by green manures were not considered, DAP: Diammonium phosphate, MOP: muriate of potash, SSP: single super phosphate, 1Beavicide®: organic pesticide containing Beauveria bassiana,2GOC: slurry made from rotten garlic, onion and chili with water, 3NeemAzal®: insecticide made from neem kernels, 4Top Ten: slurry made from leaves of ten wild plants and water, 5Verelac: organic pesticide containing Verticillium lecanii.
(DOCX)

Table S2 Detailed list of variable production costs in cotton of the farming systems compared in central India (2007–2010). ^1^ in the text, BIODYN and BIOORG are referred to consistently as organic farming systems. ^2^ in the text, CON and CONBtC are referred to consistently as conventional farming systems. ^3^ figures include time for preparation of organic fertilizers to account for their market value. ^4^ figures represent subsidized prices for mineral fertilizers set by the Government of India. ^5^ longer time required for soil cultivation in CON and CONBtC due to soil compaction. ^6^ figure includes application of biodynamic preparations. ^7^ figures include uprooting cotton and removing the straw from the field. ^8^ figures include time required to purchase inputs (organic/synthetic) from the market and to produce organic (natural) pesticides and biodynamic preparations.
(DOCX)

Table S3 Detailed list of variable production costs in soybean of the farming systems compared in central India (2007–2010). ^1^ in the text, BIODYN and BIOORG are referred to consistently as organic farming systems. ^2^ in the text, CON and CONBtC are referred to consistently as conventional farming systems. ^3^ figures include time for preparation of organic fertilizers to account for their market value. ^4^ figures represent subsidized prices for mineral fertilizers set by the Government of India. ^5^ longer time required for soil cultivation in CON and CONBtC due to soil compaction. ^6^ figure includes application of biodynamic preparations. ^7^ figures include removing soybean bundles from the field and threshing. ^8^ figures include time required to purchase inputs (organic/synthetic) from the market and to produce organic (natural) pesticides and biodynamic preparations.
(DOCX)

Table S4 Detailed list of variable production costs in wheat of the farming systems compared in central India (2007–2010). ^1^ in the text, BIODYN and BIOORG are referred to consistently as organic farming systems. ^2^ in the text, CON and CONBtC are referred to consistently as conventional farming systems. ^3^ figures include time for preparation of organic fertilizers to account for their market value. ^4^ figures represent subsidized prices for mineral fertilizers set by the Government of India. ^5^ longer time required for soil cultivation in CON and CONBtC due to soil compaction. ^6^ figure includes application of biodynamic preparations. ^7^ figures include removing wheat bundles from the field and threshing. ^8^ figures include time required to purchase inputs (organic/synthetic) from the market and to produce organic (natural) pesticides and biodynamic preparations.
(DOCX)
