# Supplementary figures and images for: Yield and Economic Performance of Organic and Conventional Cotton-Based Farming Systems – Results from a Field Trial in India
Source: PLoS One. 2013 Dec 4;8(12):e81039. doi: 10.1371/journal.pone.0081039 (PMC3852008; doi:10.1371/journal.pone.0081039)

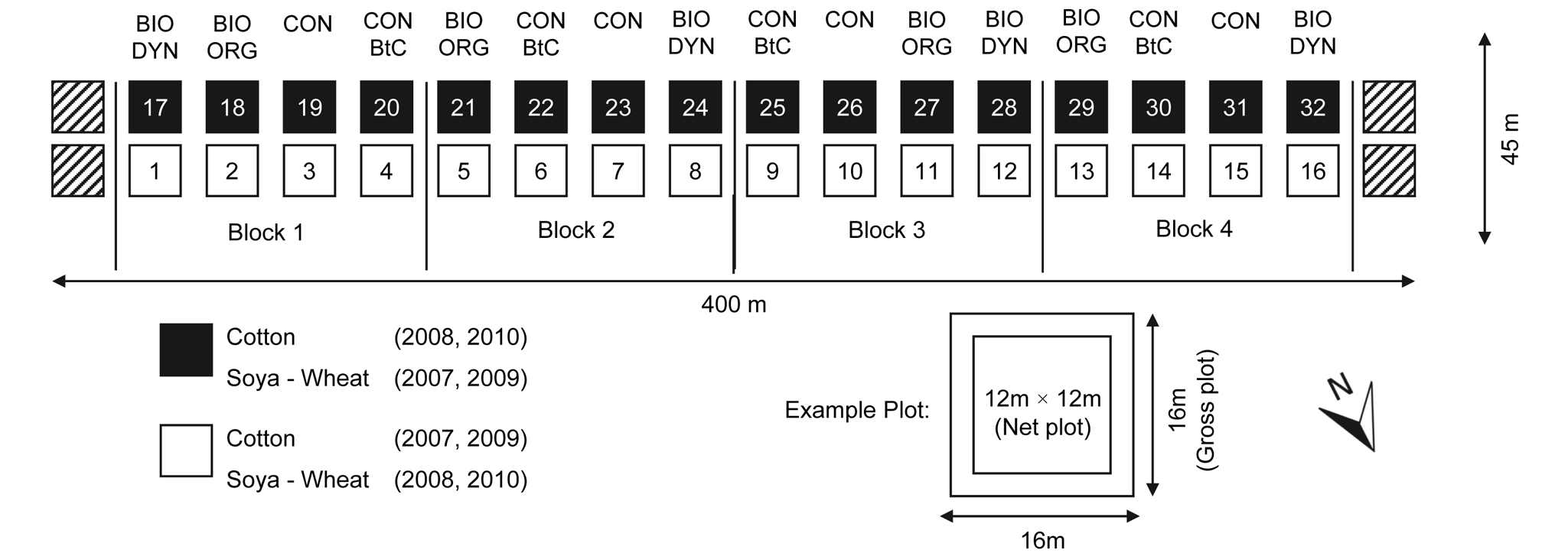

Supplement: Figure S1 — Experimental design of the farming systems comparison trial in Madhya Pradesh, India. Farming systems: biodynamic (BIODYN), organic (BIOORG), conventional (CON), conventional with Bt cotton (CONBtC), CONBtC includes wheat cultivated after Bt cotton on the same plots in 2009 and 2010, open squares belong to Strip 1, closed squares belong to Strip 2, distance between two plots within a strip = 6 m, distance between the two strips = 2 m. (TIF) [file pone.0081039.s001.tif]
