# Supplementary material for: Yield and Economic Performance of Organic and Conventional Cotton-Based Farming Systems – Results from a Field Trial in India
Source: PLoS One. 2013 Dec 4;8(12):e81039. doi: 10.1371/journal.pone.0081039 (PMC3852008; doi:10.1371/journal.pone.0081039)
Supplement: Table S1 — Fertilizer and plant protection practices in the farming systems compared in central India (2007–2010). BIODYN: biodynamic, BIOORG: organic, CON: conventional, CONBtC: conventional with Bt cotton, Ntotal: total nitrogen, OF: organic fertilizers (compost, FYM and castor cake), Ntotal includes only fertilizer derived N, nutrient inputs by green manures were not considered, DAP: Diammonium phosphate, MOP: muriate of potash, SSP: single super phosphate, 1Beavicide®: organic pesticide containing Beauveria bassiana,2GOC: slurry made from rotten garlic, onion and chili with water, 3NeemAzal®: insecticide made from neem kernels, 4Top Ten: slurry made from leaves of ten wild plants and water, 5Verelac: organic pesticide containing Verticillium lecanii. (DOCX) [file pone.0081039.s002.docx]

| Crop | Farming system | Nutrient application N_total_-P_2_O_5_-K_2_O [kg ha^-1^] | Type of fertilizers | No. of pesticide, and repellent applications (average per year) | Type of pesticides |
| --- | --- | --- | --- | --- | --- |
| Cotton | BIODYN | 105-97-208  (100% N_total_ from OF) | Castor cake, Compost, FYM | 7 | Beavicide^®1^, Butter milk, Cow Urine, GOC^2^, Biogas slurry, NeemAzal^®3^, Top Ten^4^, Verelac^®5^ |
|  | BIOORG | 105-97-208  (100% N_total_ from OF) | Castor cake, Compost, FYM | 7 | Beavicide^®^, Butter milk, Cow Urine, GOC, Biogas slurry, NeemAzal^®^-T/S, Top Ten, Verelac^®^ |
|  | CON | 146-95-88  (28% N_total_ from OF) | FYM, MOP, SSP, Urea | 7. 5 | Acephate, Endosulfan, Imidacloprid, Indoxocarb, Monocrotophos, Nitrobenzene, Profenofos |
|  | CONBtC | 166-108-138  (24% N_total_ from OF) | FYM, MOP, SSP, Urea | 7.0 | Acephate, Endosulfan, Imidacloprid, Indoxocarb, Monocrotophos, Nitrobenzene, Profenofos |
| Soybean | BIODYN | 33-27-58  (100% N_total_ from OF) | Compost, FYM | 3 | GOC, NeemAzal^®^-T/S, Top Ten |
|  | BIOORG | 33-27-58  (100% N_total_ from OF) | Compost, FYM | 3 | GOC, NeemAzal^®^-T/S, Top Ten |
|  | CON | 39-78-71  (32% N_total_ from OF) | FYM, MOP, SSP, Urea | 3.5 | Endosulfan, Imidacloprid, Monocrotophos, Nitrobenzene, Profenofos, Imazethapyr |
|  | CONBtC | 39-78-94  (32% N_total_ from OF) | FYM, MOP, SSP, Urea | 3.5 | Endosulfan, Imidacloprid, Monocrotophos, Nitrobenzene, Profenofos, Imazethapyr |
| Wheat | BIODYN | 58-56-128  (100% N_total_ from OF) | Compost, FYM | 2.5 | Cow Urine, Biogas slurry, Top Ten |
|  | BIOORG | 58-56-128  (100% N_total_ from OF) | Compost, FYM | 2.25 | Cow Urine, Top Ten |
|  | CON | 129-68-36  (4% N_total_ from OF) | DAP, MOP, SSP, Urea, FYM (only in 2007 and 2008) | 0.5 | Sulfosulfuron |
|  | CONBtC | 134-72-40  (4% N_total_ from OF) | DAP, MOP, SSP, Urea, FYM (only in 2007 and 2008) | 0.5 | Sulfosulfuron- |
