# Supplementary material for: Yield and Economic Performance of Organic and Conventional Cotton-Based Farming Systems – Results from a Field Trial in India
Source: PLoS One. 2013 Dec 4;8(12):e81039. doi: 10.1371/journal.pone.0081039 (PMC3852008; doi:10.1371/journal.pone.0081039)
Supplement: Table S2 — Detailed list of variable production costs in cotton of the farming systems compared in central India (2007–2010). 1 in the text, BIODYN and BIOORG are referred to consistently as organic farming systems. 2 in the text, CON and CONBtC are referred to consistently as conventional farming systems. 3 figures include time for preparation of organic fertilizers to account for their market value. 4 figures represent subsidized prices for mineral fertilizers set by the Government of India. 5 longer time required for soil cultivation in CON and CONBtC due to soil compaction. 6 figure includes application of biodynamic preparations. 7 figures include uprooting cotton and removing the straw from the field. 8 figures include time required to purchase inputs (organic/synthetic) from the market and to produce organic (natural) pesticides and biodynamic preparations. (DOCX) [file pone.0081039.s003.docx]

| Input / Practice | | Organic farming systems^1^ | | Conventional farming systems^2^ | |
| --- | --- | --- | --- | --- | --- |
|  | | BIODYN  biodynamic | BIOORG  organic | CON  conventional | CONBtC  conventional including Bt cotton |
| Input costs | | | | | |
|  | Seeds (incl. green manure) | 2'135 | 2'135 | 1'531 | 3'666 |
|  | Fertilizers^3,4^ | 1’285 | 1'285 | 2'941 | 3’335 |
|  | Pesticides | 997 | 997 | 3'793 | 3’527 |
|  | Irrigation fee | 456 | 456 | 456 | 456 |
| Labor costs | | | | | |
|  | Soil cultivation^5^, seed bed preparation, sowing and transplanting | 2'063 | 2'099 | 2’469 | 2’526 |
|  | Fertilizer application | 1'067 | 1'067 | 597 | 617 |
|  | Application of pesticides | 1073^6^ | 981 | 1307 | 1169 |
|  | Harvesting^7^ | 3’066 | 3’210 | 3’325 | 4’392 |
|  | Purchase and production of inputs^8^ | 828 | 661 | 712 | 650 |
|  | Weeding | 1’379 | 1’458 | 1’299 | 1’240 |
|  | Irrigation | 417 | 412 | 408 | 415 |
| Total variable costs | | 14’766 | 14’761 | 18’838 | 21’993 |
