# Supplementary material for: Yield and Economic Performance of Organic and Conventional Cotton-Based Farming Systems – Results from a Field Trial in India
Source: PLoS One. 2013 Dec 4;8(12):e81039. doi: 10.1371/journal.pone.0081039 (PMC3852008; doi:10.1371/journal.pone.0081039)
Supplement: Table S3 — Detailed list of variable production costs in soybean of the farming systems compared in central India (2007–2010). 1 in the text, BIODYN and BIOORG are referred to consistently as organic farming systems. 2 in the text, CON and CONBtC are referred to consistently as conventional farming systems. 3 figures include time for preparation of organic fertilizers to account for their market value. 4 figures represent subsidized prices for mineral fertilizers set by the Government of India. 5 longer time required for soil cultivation in CON and CONBtC due to soil compaction. 6 figure includes application of biodynamic preparations. 7 figures include removing soybean bundles from the field and threshing. 8 figures include time required to purchase inputs (organic/synthetic) from the market and to produce organic (natural) pesticides and biodynamic preparations. (DOCX) [file pone.0081039.s004.docx]

| Input / Practice | | Organic farming systems^1^ | | Conventional farming systems^2^ | |
| --- | --- | --- | --- | --- | --- |
|  | | BIODYN  biodynamic | BIOORG  organic | CON  conventional | CONBtC  conventional including Bt cotton |
| Input costs | | | | | |
|  | Seeds (incl. Rhizobia) | 2’380 | 2’380 | 2’380 | 2’380 |
|  | Fertilizers^3,4^ | 684 | 684 | 1’794 | 1’987 |
|  | Pesticides | 209 | 209 | 2’684 | 2’684 |
| Labor costs | | | | | |
|  | Soil cultivation^5^, seed bed preparation and sowing | 1’368 | 1’366 | 1’961 | 1’879 |
|  | Fertilizer application | 345 | 327 | 479 | 479 |
|  | Application of pesticides | 216^6^ | 164 | 327 | 327 |
|  | Harvesting^7^ | 1’764 | 1’757 | 2’077 | 1’961 |
|  | Purchase and production of inputs^8^ | 161 | 131 | 347 | 349 |
|  | Weeding | 368 | 359 | 288 | 279 |
| Total variable costs | | 7’495 | 7’377 | 12’337 | 12’325 |
