# Supplementary material for: Yield and Economic Performance of Organic and Conventional Cotton-Based Farming Systems – Results from a Field Trial in India
Source: PLoS One. 2013 Dec 4;8(12):e81039. doi: 10.1371/journal.pone.0081039 (PMC3852008; doi:10.1371/journal.pone.0081039)
Supplement: Table S4 — Detailed list of variable production costs in wheat of the farming systems compared in central India (2007–2010). 1 in the text, BIODYN and BIOORG are referred to consistently as organic farming systems. 2 in the text, CON and CONBtC are referred to consistently as conventional farming systems. 3 figures include time for preparation of organic fertilizers to account for their market value. 4 figures represent subsidized prices for mineral fertilizers set by the Government of India. 5 longer time required for soil cultivation in CON and CONBtC due to soil compaction. 6 figure includes application of biodynamic preparations. 7 figures include removing wheat bundles from the field and threshing. 8 figures include time required to purchase inputs (organic/synthetic) from the market and to produce organic (natural) pesticides and biodynamic preparations. (DOCX) [file pone.0081039.s005.docx]

| Input / Practice | | Organic farming systems^1^ | | Conventional farming systems^2^ | |
| --- | --- | --- | --- | --- | --- |
|  | | BIODYN  biodynamic | BIOORG  organic | CON  conventional | CONBtC  conventional including Bt cotton |
| Input costs | | | | | |
|  | Seeds | 2068 | 2068 | 2068 | 2068 |
|  | Fertilizers^3,4^ | 568 | 568 | 2’566 | 2’669 |
|  | Pesticides | 201 | 201 | 1’608 | 1’608 |
|  | Irrigation fee | 456 | 456 | 456 | 456 |
| Labor costs | | | | | |
|  | Soil cultivation^5^, seed bed preparation and planting | 1'092 | 1'108 | 1'421 | 1'407 |
|  | Fertilizer application | 648 | 620 | 353 | 345 |
|  | Application of pesticides | 254^6^ | 168 | 77 | 77 |
|  | Harvesting^7^ | 1’665 | 1’669 | 1’872 | 1’921 |
|  | Purchase and production of inputs^8^ | 225 | 219 | 356 | 346 |
|  | Weeding | 174 | 175 | 70 | 72 |
|  | Irrigation | 92 | 98 | 100 | 96 |
| Total variable costs | | 7’443 | 7’350 | 10’947 | 11’065 |
